# Qualitative Analysis of Inquiries Received by FDA Regarding Conduct of Clinical Trials during the Covid-19 Public Health Emergency

**DOI:** 10.1007/s43441-024-00674-x

**Published:** 2024-06-24

**Authors:** Marijo Kambere, Hong Vu, Dana Kappel, Kukhwa Oh, Philip Budashewitz, John Concato

**Affiliations:** 1 Office of Regulatory Affairs, Center for Drug Evaluation and Research, U.S. Food and Drug Administration, Silver Spring, US; 2 Office of Medical Policy, Center for Drug Evaluation and Research, U.S. Food and Drug Administration, 10903 New Hampshire Avenue, Silver Spring, MD WO51-6324, 20993 US; 3 Office of New Drugs, Center for Drug Evaluation and Research, U.S. Food and Drug Administration, Silver Spring, US

**Keywords:** COVID-19, Clinical trials, United States Food and Drug Administration, Drug development, Qualitative research

## Abstract

**Background:**

This report describes the U.S. Food and Drug Administration (FDA) experience in establishing a dedicated mailbox, and in publishing related guidance, to address concerns among interested parties regarding the conduct of clinical trials during the COVID-19 public health emergency (PHE).

**Methods:**

Six hundred and thirty-four mailbox inquiries were received from March 2020 through February 2022. Qualitative methods were used to provide a structured description of, and identify common themes among, these inquiries.

**Results:**

Most inquiries came from U.S.-based interested parties, including sponsors, industry trade associations, academic institutions, hospitals, clinics, research sites, trial participants, and individual persons. Approximately one-fifth of questions were related directly to COVID-19 (e.g., proposals for treatment); other inquiries were related to conduct of routine trial-related activities, and concerns were often focused on maintaining compliance with good clinical practice. In March 2020, FDA published a guidance titled *Conduct of Clinical Trials of Medical Products During the COVID-19 Public Health Emergency*; the document was subsequently revised eight times based in part on issues raised in mailbox inquiries.

**Conclusions:**

The dedicated mailbox enabled expedited communication among invested parties during the COVID-19 PHE; FDA also provided updates of the aforementioned guidance. These efforts supported the continuance of ongoing trials and the initiation of new trials during the PHE in accordance with good clinical practice guidelines, thereby helping to ensure the safety of trial participants while maintaining the quality of trial data. By soliciting and responding to trial-related inquiries and addressing corresponding needs and concerns, FDA improved transparency and communication.

## Introduction

In March 2020, and subsequent to the declaration of a public health emergency (PHE) for COVID-19 issued under Sect. 319 of the Public Health Service Act [1], the Office of Medical Policy in FDA’s Center for Drug Evaluation and Research (CDER) established a dedicated “COVID-19 mailbox” (Clinicaltrialconduct-COVID19@fda.hhs.gov) to allow interested parties to contact FDA with inquiries related to the conduct of clinical trials. In parallel, several FDA centers and offices—including CDER, the Center for Biologics Evaluation and Research (CBER), the Center for Devices and Radiological Health (CDRH), the Oncology Center of Excellence (OCE), and the Office of Good Clinical Practice (OGCP)—collaborated to issue guidance for industry, investigators, and institutional review boards (IRBs) on the *Conduct of Clinical Trials of Medical Products during the COVID-19 Public Health Emergency* [2]. The guidance document was updated multiple times (most recently in August 2021) and includes an appendix containing questions and answers based on interested parties’ input received in the dedicated mailbox.

This report summarizes findings from a qualitative content analysis of 634 inquiries received in the COVID-19 mailbox during the PHE from March 2020 through February 2022. By reviewing and providing a detailed and structured description of these inquiries, we aimed to identify common themes, gain a better understanding of the clinical trial community’s interests and concerns during the pandemic, and discuss future considerations for the post-PHE period.

## Methods

### Overview

The approaches used to analyze inquiries received in the COVID-19 mailbox were drawn from qualitative methods used in health services research, combining inductive and deductive approaches [3, 4] and using reflexive thematic analysis [5] to classify the inquiries into distinct categories. The identification of *domains* provided an overall structure to the data by classifying inquiries as studies and research focused specifically on COVID-19 studies or research, non-COVID-19 studies, or research, or not directly applicable to studies or research. Next, tags were generated by assigning concise terms to describe the distinct attributes of each inquiry. The first level of tags, reported as *topics*, were discerned from the inquiry text to provide a high-level description. Topics were based on generally accepted concepts of Good Clinical Practice (GCP) for clinical trials. For example, all inquiries that discussed managing aspects of investigational products were categorized under the same topic (i.e., “investigational product management”). Topics were further categorized into a *primary focus* and one or more *additional foci* during the data analysis phase. The coding of the primary focus (and additional foci) tags could be invoked based on a single word, a sentence, or an entire section of the inquiry transcript, as appropriate.

A deductive (general-to-specific) approach was emphasized during the process of classifying inquiries into broad domains and topics. An inductive (specific-to-general) approach was used during the process of generating primary/additional focus codes, with a focus on retaining the original intent of the inquiry. Finally, the primary and additional foci were categorized into *themes* to show broad patterns in the data. Themes were assigned by each analyst and then compared and agreed to by consensus. The themes were then ranked by frequency of occurrence, first among only the primary foci, and next with the primary and additional foci combined.

Two strategies were used to enhance the reliability of the qualitative analysis and minimize bias. First, to ensure consistency, a constant-comparison strategy was used during the analysis of the inquiries by comparing and contrasting transcripts of inquiries that had been categorized previously. Second, classifications of all inquiries were reviewed by a second classifier. More generally, the analysis adhered to Standards for Reporting Qualitative Research criteria [6].

### Source Data

The source data for this project were all inquiries received in the COVID-19 mailbox from March 2020 through February 2022. When received in the mailbox, each inquiry was given a unique identifying number, archived, and assigned to one or more FDA offices with accompanying responsibility for coordinating the development of a response. The information from the source data was stored in a structured format to track a summary of information about the inquiry, such as the date received, the issuer, a brief description of the question, and the FDA office(s) with the expertise for drafting a response. The entire original texts, as raw data, were stored separately and were the basis for conducting the current qualitative analysis. Based on a convenience sample of 193 inquiries receiving during March through September of 2020—and in the context of FDA staff prioritizing activities related to the public health emergency—the median (interquartile range) time to respond to an inquiry was 3 (2 to 5) days.

### Data Analysis

As an initial characterization of the inquiries, analysts captured the classification of sender’s affiliation (i.e., source of the inquiry), the domestic versus international affiliation of the sender, and reference to publicly available regulatory authority guidance documents.

**Stage 1** As the first review stage, familiarization with the inquiries was obtained via review of subsets of the summary and raw data, along with participation in meetings where a subset of responses to inquiries—typically those presenting more challenging regulatory issues—were discussed among multidisciplinary teams of FDA staff.

**Stage 2** A working analytical framework, consisting of preliminary tags, was developed using insights from an analyst with background in GCP and was overseen by a clinician with experience in qualitative research. During this stage, domains that make up the major structure of the data were tentatively developed, along with topics within each domain. Tags were designed to be assigned to an entire inquiry transcript and were intended to convey the main focus of each inquiry.

**Stage 3** An initial 15% sample (*n* = 95) of inquiries was selected, based on assignment to different FDA centers and offices, for developing tags that reflect the various aspects of clinical trial conduct. A primary topic was first assigned to each inquiry transcript (as described previously). Next, one or more tag(s) that capture relevant information from words, sentences, or sections of the inquiry transcripts were assigned to each inquiry to capture “primary focus” and any “additional foci.”

**Stage 4** The topics in the working analytical framework were discussed with all team members. Several iterations of the analytical framework were required before no additional topics emerged. While this research was being conducted, and based on the review of new inquiries, a limited number of new tags capturing concepts under “primary focus” and “additional foci” were added to the framework. The analytical framework was considered ready for indexing the remaining inquiries when no new concepts emerged (i.e., saturation was reached) from reviewing successive inquiry transcripts.

**Stage 5** Mapping the raw data (i.e., *n* = 634 inquiry transcripts) into the framework was ultimately performed by four analysts with backgrounds in GCP, pharmacy, epidemiology, and qualitative methods. Throughout the process of indexing and tagging the inquiries, and to document and share the analysts’ thought processes, separate notes describing the rationale for categorization were kept along with the working analytical framework. New foci were developed only when an important aspect of an inquiry was not already captured in the existing analytical framework. During the process of charting the data into the analytical framework, the team met regularly to recognize agreements, discuss discrepancies, and reach consensus.

The process of charting the inquiry transcripts into analytical frameworks was conducted in two phases, necessitated by staff availability during the COVID-19 PHE. First, the classification of inquiries received from March to May 2020 (*n* = 414 inquiries) was performed by Analyst 1 and subsequently verified by Analyst 2. An interim analytical framework was obtained when all 414 inquiry transcripts were indexed. Next, Analyst 2 applied the analytical framework to independently analyze a subset (*n* = 63) of inquiries received in the mailbox in June and July 2020 (approximately 10% of the total number of inquiries analyzed). All inquiries independently analyzed by Analyst 2 were subsequently reviewed by Analyst 1. By the end of this first interim analysis, these two analysts had analyzed a total of 477 transcripts.

A second phase of data analysis was performed by Analysts 3 and 4. These two analysts verified approximately 10% (*n* = 50) of the data indexed by Analysts 1 and 2 to become familiar with the process of charting the data into the analytical framework. Next, Analysts 3 and 4 applied the analytical framework to independently analyze the inquiries received in the mailbox from August 2020 to February 2022 (*n* = 157 inquiries; approximately 25% of the total number of inquiries analyzed). By the end of the second and final interim analysis, the four analysts had analyzed all 634 inquiries.

**Stage 6** The data classified under primary focus and additional foci were analyzed to describe the overall scope of interested parties’ inquiries. In addition, data captured under the primary focus was used to facilitate the recognition of themes and identify the main motivation for interested parties’ inquiries. Of note, all inquiries in the “miscellaneous” category (deemed non-applicable to generally accepted concepts related to clinical trial conduct and GCP) were removed from the core data analysis and were analyzed separately.

## Results

Inquiries were classified into one of seven organizational categories based on the sender’s affiliation (Table 1). More than half of senders were identified as either industry/trade association or as facilities providing health services (e.g., academic institutions, hospitals, clinics, or research sites). These organizational types, when combined, initiated one-quarter or more of the total number of inquiries.

**Table 1:** Sources of inquiries (*N* = 634 inquiries)

Organizations	# Inquiries	Percentage (%)
Industry/trade association	177	28
Academic institution/hospital/clinic/research site	156	25
Trial participant/patient/individual	120	19
CRO/CRO association	109	17
IRB/IEC	34	5
Government	33	5
Patient advocate	5	1

Inquiries were also classified into one of three categories representing domestic (U.S.), international, or unspecified origin (Table 2). Most of the inquiries (75%; *n* = 475) were identified as originating from the U.S. with others coming from international locations (11%; *n* = 88); the origin of the remaining inquiries (14%; *n* = 71) was not evident.

**Table 2:** Origin of inquiries (*N* = 634 inquiries)

Origin	# Inquiries	Percentage (%)
Domestic	475	75
Unspecified	88	14
International	71	11

The analysis of inquiry transcripts noted whether the inquiry referenced FDA or non-FDA guidance documents. A total of 129 inquiries (20%) discussed one or more topics from the FDA’s COVID-19 PHE guidance and requested further clarifications from the document. In addition, 13 inquiries (2%) referenced other regulatory authorities’ guidance related to COVID-19, and 18 inquiries (3%) referenced other FDA guidance not related to the COVID-19 PHE.

Next, we developed the analytical framework and categorization of the inquiries into topics along with primary and additional focus tags. The 16 topics (i.e., tags) that emerged included: drug or biologic approval process; device approval process; recruitment or enrollment; protocol management; obtaining or documenting informed consent; efficacy or outcome assessment; clinical records or data management; investigational product management; management of monitoring activities; clinical investigator responsibilities; IRB or Independent Ethics Committee (IEC) organization or operation; post-approval process; COVID-19-related study or research; COVID-19-related therapy or prevention; patient or COVID-19 vulnerable individual; and miscellaneous. The topics listed were used to further expand in creating the primary and additional foci of the inquiries (Table 3).

**Table 3:** Working analytical framework (*N* = 634 inquiries)

Domain (preliminary tag)	Primary or additional foci
Guidance documents	Issuance of COVID-19-related regulatory guidelines
Obtaining or documenting informed consent	Informed consent process; content; documentation
Protocol management	Study or protocol amendment change; deviation handling
	Study or protocol design
	COVID-19 study participation
	Study eligibility; screening procedures
	Virtual site visits/telehealth
	New or alternative clinical site
	Human specimen handling
	Study delay; suspension; premature termination
	Study resumption after temporary pause
Investigational product management	Direct-to-patient distribution of IP
	Home or alternate site for infusion/injection
	Commercial procurement; supply of IP
	Suspension/premature termination of IP
	Over-the-counter products marketing
	Diagnostic test validation; certification; marketing
	COVID-19 test validation; certification; marketing
	COVID-19 vaccine; treatment proposal
	cGMP or labelling compliance
	Prescription for compounded medication; placebo
	Personal Protective Equipment (PPE) validation; certification; marketing
	Chemical or non-chemical disinfectant
Efficacy or outcome assessment	Altered efficacy endpoint; primary outcome assessment
	Adverse events handling; reporting
	COVID-19 diagnostic test result handling; safety reporting
	Bioequivalence; bioavailability assessment
Clinical records or data management	Electronic signature; record; system compliance
	Clinical data; statistical analysis
	End of study database lock handling
	Lost or contaminated source document
Clinical investigator responsibilities	New or alternative Investigator
IRB or IEC organization or operation	Informing or reporting to IRB
	IRB operation; review process
	Virtual IRB; IEC meeting
Management of monitoring activities	Remote clinical data monitoring
	Wearable device; mobile health data handling
	FDA Inspection during pandemic
Informing or interacting with FDA	Repurposing; treatment IND; emergency use process
	COVID-19 trial regulatory approval process
	Informing or interacting with FDA; reporting; submission process
	Informing or interacting with FDA; meeting; call; POC
	Informing or interacting with FDA; alternate approach to submission
	Reporting of trial misconduct
	Waiver or exemption
	Expedited approval; review process
	User fee review activities

Data captured under the foci-related categories were used to group the inquiries into themes. We analyzed 572 inquiries after removing 10% (*n* = 62) of the inquiries from the analysis due to being classified as lacking direct relevance to clinical trials, COVID-19 studies, or COVID-19 products or treatment. The themes (*n* = 22) are listed in Table 4 and reflect the broad interest of interested parties. For example, approximately 21% (*n* = 120) of the primary foci pertain to COVID-19 related studies, research, or treatment proposals. Inquiries related to informed consent, system compliance, remote monitoring, and interacting with FDA were also commonly encountered (approximately 8% for each theme). Representative examples of the five most common themes include:

**Table 4:** Ranking of themes based on primary focus (*N* = 572 inquiries)

Rank	Primary focus theme	# Inquiries	Percentage (%)
#1	COVID-19-related study/research/treatment proposals	120	21
#2	Informed Consent process/content/documentation	47	8
#3	Electronic signature/record/system compliance	47	8
#4	Remote data monitoring/wearables/mobile data health handling	46	8
#5	Informing/interacting with FDA	45	8
#6	Study eligibility/screening procedures/COVID-19 study participation	38	7
#7	IP distribution/supply/suspension	34	6
#8	Study/protocol amendment/change/deviation handling	27	5
#9	IRB/IEC organization/operation	21	4
#10	New/alternative clinical site/investigator	20	4
#11	Issuance of COVID-19-related regulatory guidelines/resources	20	4
#12	AE handling /safety reporting	19	3
#13	Study delay/suspension/premature termination/resumption after pause	18	3
#14	Study design/regulatory approval and process including digital tools	16	3
#15	Virtual site visits/telehealth	16	3
#16	Clinical data/statistical analysis /end of study database lock	10	2
#17	FDA inspection during pandemic	8	1
#18	cGMP/labelling compliance	7	1
#19	Altered efficacy endpoint/primary outcome assessment	5	1
#20	Bioequivalence/bioavailability assessment	4	1
#21	Human specimen handling	3	1
#22	User fee review activities	1	0.2

- COVID-19-related study/research/treatment proposals
  - “I was wondering if there is a list available of possible treatments that are currently reviewed by FDA under the [Coronavirus Treatment Acceleration Program]?”
- Informed Consent process/content/documentation
  - Do we need an impartial witness if we are doing the informed consent process over the phone and are able to get a copy/scan/photograph of the subject’s signed signature page?”
- Electronic signature/record/system compliance
  - “Does the FDA offer an appropriate solution for finalization of study related document that are typically provided as wet signatures? Would it be acceptable for clients to provide [a] photograph of the page with their signature in the interim until the page with wet signatures are scanned or mailed to the study lead?”
- Remote data monitoring/wearables/mobile data health handling
  - “If a site has paper source documents for subjects which need to be reviewed prior to monitoring verification in an [electronic data capture system], but the site is not allowing on-site monitoring visits due to the COVID-19 pandemic– what guidance, if any, does the FDA provide for potential remote verification?”
- Informing/interacting with FDA
  - “We have a clinical trial with an [Investigational New Drug Application]. In response to the COVID-19 pandemic our [Institutional Review Board *at hospital site*] has requested us to stop all new recruitment and in-person study related visits. My question is, are we required to inform the appropriate FDA division of this change in study status.”

After the analysis of the primary focus of each inquiry, an analysis of the primary focus (*n* = 572) plus additional foci (*n* = 298) was completed (Table 5), representing a total of 870 foci. Overall, the top ten inquiry themes in Tables 4 and 5 were generally similar.

**Table 5:** Ranking of top ten inquiry themes based on primary and additional foci (*N* = 870 Foci)

Rank	Primary and additional focus theme	# Foci	Percentage (%)
#1	COVID-19-related study/research/treatment proposals	171	20
#2	Informing/interacting with FDA	94	11
#3	Informed consent process/content/documentation	73	8
#4	Remote data monitoring/wearables/mobile data handling	61	7
#5	Study/protocol amendment change/deviation handling	57	7
#6	Electronic signature/record/system compliance	49	6
#7	IRB/IEC organization/operation	43	5
#8	IP distribution/supply/suspension	42	5
#9	Study eligibility/screening procedures/COVID-19 study participation	40	5
#10	Access to/issuance of COVID-19-related regulatory guidelines /resources	39	5

Representative details regarding inquiries and corresponding responses are described in this report; the responses (not discussed herein) were also used to inform updates of the FDA guidance on clinical trial conduct during the COVID-19 PHE. That guidance document was updated eight times, most recently in August 2021, including via the addition and sequential expansion of an appendix containing questions and answers based on interested parties’ input received in the dedicated mailbox.

The most common focus of inquiries involved COVID-19-related research, studies, or treatment proposals. Given that the focus of the mailbox and corresponding guidance was on clinical trial conduct in general, such inquiries were forwarded to appropriate FDA offices. Also of note, the COVID-19 mailbox is only one of various mailboxes created by FDA regarding the COVID-19 PHE, and this report does not describe nor discuss all inquiries about drug development programs received during the PHE.

Next in frequency, inquiries pointed to uncertainty among interested parties on how and whom to contact at the FDA. Interested parties usually requested specific contact information to receive FDA input on protocol amendments, deviations, and data management activities. As a result, the FDA provided insights on existing FDA procedures, facilitated connections with appropriate internal contacts, and updated the COVID-19 guidance with related information that allowed the sponsors to make informed decisions [2].

The third most frequent type of inquiry was regarding the informed consent process during the COVID-19 PHE. Traditionally, the informed consent process starts when a patient goes to a clinical trial site for in-person discussions regarding risks and benefits of clinical trial participation [7]. Given that the COVID-19 PHE led to new limitations—such as quarantine or social distancing with restrictions on face-to-face interaction, resulting in an inability for clinical trial administrators and trial participants to travel to trial sites [8]—interested parties had many questions regarding the informed consent process. Several questions and answers in the COVID-19 guidance were added based on these inquiries, as a representative example of the link between the mailbox and the guidance [2]. Not surprisingly, questions related to electronic systems used to generate electronic signatures on clinical trial records, including informed consent documents, form a common theme and these questions ranked within the top five Primary Focus themes and the top six Primary and Additional Focus themes. Of note, a related question and answer were included in the Appendix of the guidance document [2].

The fourth most frequent inquiries were related to remote assessments and related technology-assisted remote methods of data collection and monitoring. Many clinical trials include decentralized aspects, such as laboratory tests conducted at facilities other than the site where in-person medical assessments are conducted, and the benefits of these practices were promoted during the PHE to assure patient safety in conjunction with social distancing restrictions. For example, ongoing and new trials replaced traditional safety monitoring at on-site visits with telephone or video visits. The COVID-19 guidance document provided general considerations regarding remote clinic visit and clinical outcome assessments, remote site monitoring visits, and the use of technology systems as well as technological tools that support remote trial data acquisition and analyses [2].

The fifth most frequent inquiries were regarding changes, deviations, and amendments to studies. Early during the PHE, FDA and sponsors of clinical trials recognized that some protocol changes necessitated by the impact of COVID-19 would be unavoidable, and this issue was therefore one of the first topics discussed during the development of the body of the guidance document and associated Appendix [2].

Sixty-two inquiries were categorized as “miscellaneous” due to a lack of direct relevance to clinical trials, COVID-19 studies, or COVID-19 treatment; see Table 6. Of note, 38 inquiries were categorized as miscellaneous despite asking about COVID-19-related FDA general resources/materials, given that the corresponding questions did not focus on clinical trial conduct during the COVID-19 pandemic. Six inquiries were related to COVID-19-related study funding and five were related to marketing.

**Table 6:** Detailed tags of inquiries removed from the analysis (*N* = 62 inquiries)

Miscellaneous	# Inquiries	Percentage (%)
Access to COVID-19-related FDA resources/materials	38	61
COVID-19-related study funding	6	10
Access to non-COVID-19-related FDA resources/materials	5	8
Guidelines on social distancing/lockdown/wearing PPEs	5	8
Marketing for digital/electronic tools for health-related services/information	3	5
Marketing for COVID-19-related R&D facility	1	2
Marketing for COVID-19-related online portal/resources	1	2
Perform data collection/entry for FDA to expedite review process	1	2
Defining FDA-regulated medical device	1	2
Healthcare provider payment reporting guidelines	1	2

## Discussion

When the U.S. Secretary of Health and Human Services declared a public health emergency [1], FDA recognized the potential for major disruptions in the conduct of clinical trials. As such, a guidance [2] on clinical trial conduct and a linked COVID-19 mailbox were promptly created to provide interested parties with information and a mechanism to contact FDA with inquiries related to clinical trials regulations and procedures. The PHE placed time pressures on the FDA to develop prompt policy responses. The ability to rapidly publish and then update the COVID-19 trial guidance to address the COVID-19 PHE was permitted under Sect. 319 of the PHS Act [9]. This Act allows the FDA to implement a range of policies and regulations without prior public comment, but subject to comment after publication in accordance with the Agency’s good guidance practices [10, 11]. The COVID-19 guidance provided real-time information to assist sponsors, IRBs, and clinical investigators in assuring the safety of trial participants, maintaining compliance with GCP, and minimizing risks to trial integrity [2].

The guidance document, mailbox, and responses to queries were mechanisms that allowed the Agency to address challenges in conducting clinical trials posed by the PHE. Updates to the guidance primarily included the addition and expansion of an appendix containing questions and answers based on input from interested parties received in the dedicated mailbox. By adopting a proactive and collaborative approach, the FDA enabled efficient handling of inquiries and provided interested parties and the public with consistent and timely messaging.

The FDA also recognized that the use of technologies, such as mobile devices, and internet portals or applications, facilitates the ability to collect more trial elements remotely and provides innovative ways for conducting clinical studies. As such, and initiated prior to the PHE, a guidance document was published in December 2021 (during the PHE) that provided recommendations to sponsors, investigators, and other interested parties on the use of digital health technologies (DHTs) to acquire data remotely from participants in clinical investigations [12]. The COVID-19 and DHTs guidance documents also support decentralized trials by facilitating interactions with trial participants in locations other than a traditional clinical trial setting, an important aspect in a PHE response. In addition, decentralized trials have the potential to increase trial participant diversity and improve trial efficiencies by reaching targeted populations [13, 14]. The FDA guidance on decentralized trials was published in May 2023 [13].

The COVID-19 mailbox, inquiries from interested parties, and the subsequent creation of the COVID-19 clinical trial guidance also has provided an opportunity to apply the knowledge gained and lessons learned during the pandemic to future guidance and to open further discussion with interested parties regarding PHEs. After the HHS Secretary’s announcement that the PHE declaration expired on May 11, 2023, the FDA *Guidance on Conduct of Clinical Trials of Medical Products During the COVID-19 PHE* was revised and republished for future disasters and PHEs that may lead to disruption in clinical studies [15, 16, 17]. This FDA guidance, titled *Considerations for the Conduct of Clinical Trials of Medical Products During Major Disruptions Due to Disasters and Public Health Emergencies*, reflects the Agency applying lessons learned from the COVID-19 PHE. The guidance provides a framework for sponsors of clinical trials regarding how to adopt the contingency planning in clinical trials and highlights strategies for maintaining data quality and integrity during major trial disruptions [17].

In response to the Food and Drug Omnibus Reform Act (FDORA) requirements [18], FDA is conducting several public workshops to solicit input from industry and other interested parties on diverse topics related to the conduct of clinical trials, such as mitigating clinical trial disruptions and increasing diversity in clinical trials [19, 20, 21]. For example, the FDA in collaboration with the Clinical Trials Transformative Initiative held a public workshop in October 2023 titled “Mitigating Clinical Study Disruptions During Disasters and Public Health Emergencies” [21]. The workshop discussed recommendations provided by the FDA during the COVID-19 PHE to mitigate disruption of clinical studies and included sessions with invested parties to gain ideas to prepare for future PHEs [21]. Representatives from the FDA, industry and academic sponsors, study teams, and patient advocacy groups shared their experiences and best practices learned while navigating disruptions in clinical studies during the COVID-19 PHE, as they relate to preparedness and response capabilities. These activities continued the important collaboration between the FDA and interested parties that started with the COVID-19 mailbox and COVID-19 clinical trial guidance.

## Conclusions

The results from this research provide a foundation to create a streamlined process of communication in a public health emergency. This report describes how the FDA addressed the COVID-19 PHE while adhering to existing regulations governing human subject protection and the conduct of clinical trials. Establishment of a dedicated mailbox for questions related to clinical trial conduct enabled communication among FDA, sponsors, and other interested parties during the COVID-19 PHE. Along with related guidance, FDA was able to describe and clarify agency policies that facilitated the continuance of ongoing trials and the initiation of new trials—thereby promoting the safety of trial participants and helping maintain compliance with good clinical practices, while lessening risks to trial integrity. By monitoring and analyzing interested parties’ clinical trial-related inquiries, we were better able to identify their needs, address their concerns, and improve transparency in communication.

## Data Availability

Data that support the findings of this study represent information held internally at the U.S. Food and Drug Administration.

## Declarations

### Disclaimer

This article reflects the views of the authors and should not be construed to represent FDA’s views or policies.
